# The Effectiveness of Pay-for-Performance Program of Diabetes Care for Psychiatric Patients: A Regional Psychiatric Hospital Experience

**DOI:** 10.3390/healthcare9111565

**Published:** 2021-11-17

**Authors:** Chin-Chou Yang, Tsuo-Hung Lan, Wei-Cheng Tsai, Ming-Chen Guo, Yee-Yung Ng, Shiao-Chi Wu

**Affiliations:** 1 Institute of Health and Welfare Policy, National Yang Ming Chiao Tung University, Taipei 112, Taiwan; ttpcabay@gmail.com (C.-C.Y.); ethanwctsai@nycu.edu.tw (W.-C.T.); tea894301@gmail.com (M.-C.G.); 2 Tsao-Tun Psychiatric Center, Ministry of Health and Welfare, Nantou 542, Taiwan; tosafish@ttpc.mohw.gov.tw; 3 Department of Medicine, National Yang Ming Chiao Tung University, Taipei 112, Taiwan; 4 Center for Neuropsychiatric Research, National Health Research Institutes, Miaoli 350, Taiwan; 5 Institute of Clinical Medicine, National Yang Ming Chiao Tung University, Taipei 112, Taiwan; 6 Department of Medicine, Fu Jen Catholic University Hospital, Fu Jen Catholic University, New Taipei City 243, Taiwan; yyngscwu12@gmail.com

**Keywords:** diabetes, mental illness, Pay-for-performance

## Abstract

Background: The Pay-for-performance (P4P) program of diabetes care has demonstrated successful outcomes in patients with type 2 diabetes. However, the effectiveness of this multidisciplinary care model for psychiatric patients has never been evaluated. The objective of this study is to examine the effectiveness of P4P program of diabetes for psychiatric patients with diabetes. Methods: This study utilized a retrospective cohort design to examine the effectiveness of P4P program of diabetes care for psychiatric patients with diabetes. The participants’ HbA1c (hemoglobin A1c) data of the fourth quarter in 2018 were used as baseline value, while P4P program was not applied yet. HbA1c data of every quarter in 2019 were collected. Generalized estimating equations (GEE) was used to analyze the change of HbA1c level. Results: The HbA1c level increased slightly in the first quarter, and then decreased gradually since the second quarter. The HbA1c level was significantly lower in the fourth quarter after P4P program intervention (*p* < 0.05). Conclusion: P4P program of diabetes care is also effective on psychiatric patients with diabetes, and this multidisciplinary care model could be encouraged and promoted for psychiatric patients with diabetes.

## 1. Introduction

Diabetes is a common health issue in the world. The prevalence of diabetes in patients with mental illnesses, such as schizophrenia, bipolar disorder, and anxiety disorder, are higher than general population [1,2,3,4]. The mental disorders have adverse effects on diabetes control [5].

Since Pay-for-performance (P4P) was introduced in UK primary care, based on the concept of the Quality and Outcomes Framework (QOF) in April 2004 [6], P4P programs have been widely adopted in many countries to improve the quality of healthcare, especially for chronic diseases [7]. P4P is a cost-effective policy to stimulate improvements in healthcare efficiency and quality by financial incentives, as this payment model rewards health care providers if they meet defined indicators or parameters to increase the quality or efficacy of care.

Good diabetes control has already been proven efficacious to prevent many acute and chronic diabetic complications [8]. The P4P program of diabetes care, which was initiated in 2001 by the Ministry of Health and Welfare in Taiwan, helps patients with diabetes to achieve better glycemic control [9]. This program is an important health policy designed to prevent diabetes-related complications. The participating physicians, nurses, and dietitians are required to have specialized training for diabetes care. The patient-centered care model focuses on diabetes self-management education, better adherence to instructions, and screening for diabetes-related complications (such as retinopathy, neuropathy, and cardiovascular complications). This care model has demonstrated successful outcomes in the P4P participants with type 2 diabetes [10]. However, there were no endocrinologist hired by any psychiatric hospital in Taiwan before 2019, and the P4P program of diabetes has never been adopted in psychiatric hospitals.

Taiwan established national health insurance (NHI) in 1995, and the coverage of NHI is 99.9%. Nearly everyone in Taiwan is covered by NHI, including those with mental disability. Up to 2017, according to the National Statistic Report of Ministry of Health and Welfare in Taiwan, there were about 1,167,000 people certified with mental disability. Most of them were living in the community. However, for some patients who have severe mental illness, they may reside in chronic wards of psychiatric hospitals. Most of these patients are certified as catastrophic illness with copayment exemptions. The psychiatric hospital is a special system in Taiwan. These hospitals not only provide services of admission and emergency treatment for psychiatric problems, but also have out-patient departments providing services for patients with mental illness. Beside treatment for mental illness, patients may also receive treatment for diabetes from psychiatrist at the same time.

The combination of mental illness and diabetes is related to poor health outcomes [11]. However, caring for psychiatric patients with diabetes is a complex process that needs to consider the emotional and psychological aspects as well as metabolic control at the same time. In 2019, the P4P program of diabetes care was implemented in a regional psychiatric hospital, which was also the only psychiatric hospital that hired a full-time endocrinologist in Taiwan. This regional psychiatric hospital had about 1200 psychiatric beds, including outpatient’s clinic, emergency department, intensive care unit, 6 acute and 16 chronic wards, and 4 nursing homes. The objective of this study is to evaluate the effectiveness of the P4P program of diabetes for outpatients and assess the quality of care and outcome of psychiatric patients with diabetes.

## 2. Materials and Methods

Our study utilized a retrospective cohort design to examine the effectiveness of P4P program of diabetes care for psychiatric patients with diabetes in the outpatient department of a regional psychiatric hospital in middle Taiwan. An endocrinologist was hired in 2018 to provide metabolism clinic service in this psychiatric hospital. Before that, some patients may accept treatment for diabetes and mental illness together in the psychiatry department, and some patients may seek treatment for diabetes in other medical hospitals. Since then, psychiatric patients with diabetes were referred to the endocrinologist. Before 2019, the endocrinologist provided diabetes treatment alone. The process of delivering diabetes care not only requires professional skills, but also requires a multidisciplinary approach, especially for psychiatric patients. According to the rule of the P4P program of diabetes care, physicians, nurses, and dieticians need to accept specialized training programs before participating in the program. After all team members finished the training program for diabetes care (including 96 h of lectures and 80 h of practical courses), the hospital was certificated as a qualified medical facility to perform P4P program in January 2019.

The staff members of the P4P program worked as a coordinated multidisciplinary team, which consisted of a full-time endocrinologist, diabetes nurses, and dieticians. The endocrinologist emerged as the leader, coordinating every team member to assure that all treatments met the clinical guidelines established for the care of diabetes patients. The care team was required to provide regular evaluations for the enrolled patients, including biochemical tests, educational programs, and management plans. In addition to regular out-patients service fees, hospitals received TWD (New Taiwan Dollar) $650 (US$23.36) per initial enrollment visit, TWD $200 (US$7.19) per follow-up visit, and TWD $800 (US$28.75) per annual evaluation visit. Additional incentive based on two positive indicators (percentage of HbA1c (hemoglobin A1c) <7%, percentage of completed follow-up visits) and two negative indicators (percentage of HbA1c >9.5%, percentage of LDL (low-density lipoprotein) >130 mg/dL) were also provided. Because all hospitals participating in the P4P program were required to submit data to Virtual Private Network of NHI for P4P follow-up payments, our database is undoubtedly reliable.

**Figure 1:**
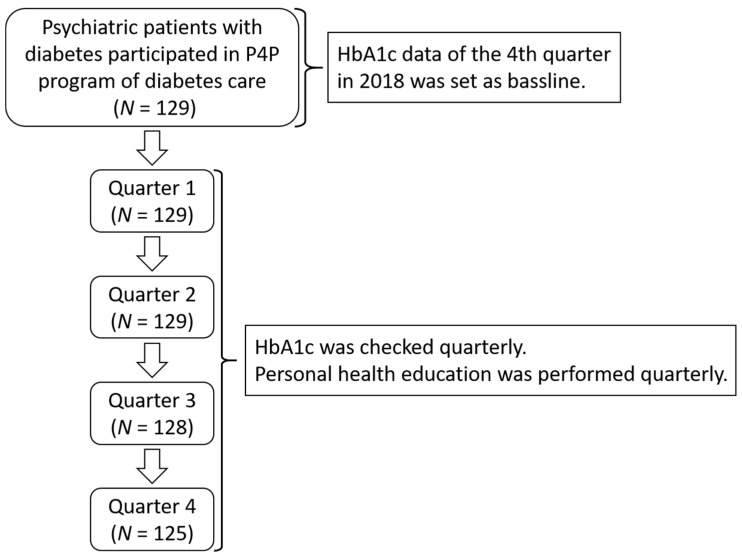
Flow diagram of our study. A total of 129 psychiatric patients with diabetes were included in the beginning. One patient loss to follow-up in the third quarter, and another three patients loss to follow-up in the fourth quarter. P4P: pay-for-performance; HbA1c: hemoglobin A1c.

The study selection process is illustrated in Figure 1. In the first quarter of 2019, all of the 129 psychiatric patients with diabetes who accepted treatment in the endocrinology (clinic) in this psychiatric hospital participated in P4P program. Their hemoglobin A1c (HbA1c), a diagnostic and care-quality indicator of diabetes, were checked and collected every 3 months. In clinical practice, it was usually hard to clarify whether patients with mental illness actually received fasting measurement or not. HbA1c does not require fasting and can be offered to non-fasting patients. Individualized health education was also provided every 3 months. We collected the participants’ HbA1c data of the fourth quarter in 2018 as the baseline value, while the P4P program was not applied yet. The basic characteristics of our patient, including sex (1 = male, 0 = female), age, body mass index (BMI), and comorbidities (hypertension and hyperlipidemia; 1 = yes, 0 = no) were recorded as covariance. Generalized estimating equations (GEE) can take into account the correlation of within-subject data (longitudinal studies) and other studies in which data are clustered within subgroups [12]. This study was a repeated-measure design to predict change in the HbA1c level of the individual over 3, 6, 9, and 12 months from the baseline. In order to avoid bias resulting from the correlation of within-subject, which may result in a wrong inference result, this study conducted GEE, which provided the population-averaged estimates of the parameter for statistical analysis. Sex (1 = male, 0 = female) and comorbidities (1 = yes, 0 = no) were set as categorical variables. HbA1c level, age, and BMI were set as continuous variables. Dummy variables were used to represent follow-up (0 = baseline, 1 = 3 months, 2 = 6 months, 3 = 9 months, 4 = 12 months). The statistical analysis was done using SPSS 26 (IBM, Armonk, NY, USA).

## 3. Results

There were 129 patients enrolled in our study. A total of 53.5% were male, and 46.5% were female. The prevalence of hypertension and hyperlipidemia were 51.2% and 79.8%. The mean HbA1c level was significantly higher in female patients than in male patients (*p* < 0.05). The prevalence of hypertension was lower among male patients (49.3%) than female patients (53.3%) (*p* > 0.05). The prevalence of hyperlipidemia was slightly higher among male patients (84.1%) than female patients (75.0%) (*p* > 0.05) (Table 1).

The HbA1c level increased slightly in the first quarter (3 months), and then decreased gradually after the second quarter (6 months). In Model 1, before adjusting covariance, the HbA1c level was significantly lower in the third quarter (9 months) (*p* < 0.05), and the effectiveness of P4P persisted to the fourth quarter (12 months) (*p* < 0.05). In Model 2, after controlled covariance of age, sex, and BMI, the HbA1c level was significantly lower in the fourth quarter (*p* < 0.05). This result was also found in Model 3, after we added comorbidities (hypertension and hyperlipidemia) as the covariance (*p* < 0.05). The effectiveness of P4P intervention on the mean HbA1c level was better in patients without hyperlipidemia than in those with hyperlipidemia (*p* < 0.05), as shown in Table 2.

## 4. Discussion

To the best of our knowledge, this is the first study evaluating the effectiveness of the P4P program of diabetes care for psychiatric patients in Asia. This study also supported the previous report that HbA1c level was significantly lower after P4P program intervention. The effective period was longer than that for the general population.

People with mental illness have a higher risk of developing diabetes mellitus and its following complications [13]. For patients with coexisting mental illness and diabetes, it is difficult to manage their blood sugar levels [14]. Although it has been reported that diabetes care programs can lead to better health outcomes, there were few practical and effective care models applied for people with mental illness. Lower quality of medical care for psychiatric patients with diabetes might result in poor diabetes outcomes [15]. The clinical management of mental disorders and diabetes may include many drugs. Adverse effects of certain medication may cause weight gain and increase the risk of diabetes [2]. Besides, psychiatric patients may have barriers at the personal, social, and health care system level [16]. These problems are significant challenges in managing diabetes for patients with mental illness. In our study, the implementation of the P4P program of diabetes provided effective management of diabetes mellitus for patients with mental illness. With multidisciplinary care, patients receiving treatment for mental and metabolic disorder in the same regional psychiatric hospital may increase their adherence to regular therapy by saving time and money. A previous study also demonstrated that patients participating in the P4P program of diabetes care may have better medical adherence [17]. Besides, P4P is a financial incentive program based on quality indicators such as complete patient follow-up, level of HbA1c, and lipid profile. Hence, the P4P program may also help care-team members to monitor patients’ condition more carefully. These factors may improve the quality of diabetes care for patients with mental illness.

The success of the P4P program for psychiatric patients may also result from the multidisciplinary care design. Multidisciplinary approach to diabetes care was associated with better glycemic control and quality care [18]. Patients with mental illness may have cognitive impairment, which can disrupt attention and learning. Such characteristics may make it difficult to get information about diabetes or other medical problems. The multidisciplinary care model may help these patients accept recommendations from care-team members to achieve goals of diabetes management. Besides regular physical examination and laboratory evaluation, diabetes self-management education is also a quality indicator of the P4P program of diabetes care. Diabetes education is an important element of diabetes control [19]. During the process of education, strategies were provided to patients and their families, helping them understand how and what they should do to improve patient’s health condition. Education of P4P program promoted self-management and helped patients to engage in healthy behaviors that improve diabetes control. In the P4P program, participating physicians and qualified diabetes educators must provide regular patient-centered care education, including lifestyle and diet changes, sugar monitoring, and taking regular medications. These self-management behaviors helped patients to have a better quality of diabetes care.

Because of the improvement of glycemic control [10,20], the frequency of diabetes-related hospitalization rates and medical costs [21] may also be decreased. Our study showed that male patients were more likely to benefit from the P4P program of diabetes, but the statistics are not significant. This finding can be explained by previous research [22], where male patients seemed to have stronger perceptions of the need to control diabetes and better social support than female patients. Besides, the baseline HbA1c level was higher in female patients. These factors may explain the sex differences in glycemic control in our study.

In our study, the slight increase of HbA1c level in the first quarter may be related to the new year and lunar new year festivals in January and February. The heavy meals, sweet desserts, and less exercise in the above festivals may make the blood sugar level higher than other seasons. The HbA1c level was significantly lower in the third quarter in Model 1, and in the fourth quarter in Models 2 and 3. This effective period was longer than previous research in Taiwan [23]. Hsu et al. study found that the HbA1c level in the P4P group was significantly lower than that in the control group, beginning at the sixth month. The longer effective period may be related to the complex set of social circumstances and personal condition of psychiatric patients. Although patients with mental illness might take longer to improve their sugar control under P4P program, this study confirmed the effectiveness of P4P program on blood sugar control in psychiatric patients with diabetes.

Dyslipidemia is commonly seen in diabetic patients [24]. In diabetic patients, some lipoprotein abnormalities may lead to peripheral insulin resistance and poor glycemic control [25]. Besides, obesity could also induce insulin resistance. A direct relationship between BMI and diabetes has been demonstrated [26]. The prevalence of dyslipidemia among people with mental illness is higher than the general population [27], which might make diabetes control more difficult. Additionally, people with mental illness are likely to be overweight or obese [28], which leads to worse glycemic control. These factors may explain the results of our study that the HbA1c level seems to be poorly controlled in patients with hyperlipidemia and higher BMI.

The incentive payments (additional TWD $650 (US$23.36) per initial enrollment visit, TWD $200 (US$7.19) per follow-up visit, and TWD $800 (US$28.75) per annual evaluation) of the P4P program from National Health Insurance may encourage hospitals to support and set up the P4P care team. These incentive payments and positive indicators may play an important role in maintaining the care team, achievement, good relationship between patients and team members, and increase the intrinsic motivation of health professionals. In consequence, the program acted as a booster to enhance the quality of diabetes care for psychiatric patients.

There were limitations in our study. First, the results from the limited number patients and one regional hospital may need a further case control study for support. Second, before the P4P program, some patients had received treatment from the same endocrinologist in this study for one year. It might not be easy to clarify whether the improvement of the HbA1c level is the natural course of the endocrinologist’s one-year treatment effect or the effect of the P4P program. However, if it was the natural course of the endocrinologist’s one-year treatment effect, the HbA1c level should be significantly lower from the first quarter of P4P program, not from the third to the fourth quarter of P4P program. Therefore, the effectiveness of P4P program on blood sugar control in psychiatric patients is worth to be encouraged. Third, we did not ask patients to quantify the change of biophysical parameters, such as diet or behavior change to assess its effects on sugar control when considering the inaccuracy of psychiatric recording. This limitation might be compensated by the diet education of the P4P program. In clinical practice, team members provided individualized treatment and education program for each patient. Team members may spend more time discussing or educating patients with poorer controlled diabetes or severe psychiatric conditions. There were only four patients that were lost in the follow-up by the end of this study, which may support the notion that most patients were compliant with the P4P program.

## 5. Conclusions

This study showed that the P4P program for diabetes care is also effective on psychiatric patients with diabetes. The patients’ HbA1c level was significantly lower after P4P intervention. The P4P program of diabetes care could be encouraged and promoted for psychiatric patients with diabetes in order to reduce the adverse effects of poor sugar control.

## Figures and Tables

**Table 1:** Basic characteristics of 129 psychiatric patients with diabetes.

Characteristics	Total (*n* = 129)	Male (*n* = 69)	Female (*n* = 60)	*p*-Value
	Mean ± SD	Mean ± SD	Mean ± SD	
Age	55.08 ± 13.18	53.28 ± 12.82	57.14 ± 13.39	0.10
BMI	26.99 ± 5.07	26.19 ± 4.32	27.93 ± 5.74	0.08
HbA1c	6.82 ± 1.31	6.58 ± 0.96	7.09 ± 1.58	0.03
Hypertension *n*	(66, 51.2%)	(34, 49.3%)	(32, 53.3%)	0.65
Hyperlipidemia *n*	(103, 79.8%)	(58, 84.1%)	(45, 75.0%)	0.20

BMI: body mass index; HbA1c: hemoglobin A1c; SD: Standard Deviation.

**Table 2:** Change in the HbA1c levels of diabetic psychiatric patients after implementing P4P intervention for one year.

Items	HbA1c	Model 1			Model 2			Model 3		
	Mean (SD)	β	se	*p*	β	se	*p*	β	se	*p*
Follow-up										
Baseline (ref = 0)	6.82 (1.31)									
3 months	6.89 (1.54)	0.07	0.09	0.42	−0.02	0.07	0.78	−0.02	0.07	0.78
6 months	6.64 (1.29)	−0.18	0.10	0.08	−0.18	0.12	0.13	−0.18	0.12	0.13
9 months	6.58 (1.21)	−0.23	0.11	0.03	−0.24	0.13	0.06	−0.24	0.13	0.06
12 months	6.50 (1.22)	−0.32	0.11	<0.01	−0.31	0.13	0.02	−0.31	0.13	0.02
Age					0.01	0.01	0.37	0.01	0.01	0.21
Sex (ref = female)					−0.23	0.22	0.29	−0.30	0.21	0.16
BMI					0.04	0.02	0.09	0.02	0.03	0.35
Hypertension (no = 0)								0.03	0.22	0.90
Hyperlipidemia (no = 0)								0.76	0.18	<0.01

In order to avoid bias resulting from the correlation of within-subject, which may result in wrong inference result, this study conducted generalized estimating equations (GEE), which provided the population-averaged estimates of the parameter for statistical analysis. Using the GEE to predict change in HbA1c level of the individual over 3, 6, 9, and 12 months from baseline. HbA1c level, age, and BMI were set as continuous variables. SD: Standard Deviation; BMI: Body Mass Index; HbA1c: hemoglobin A1c; ref = reference.

## Data Availability

Not applicable.

## References

[1] Grigolon R.B., Brietzke E., Mansur R.B., Idzikowski M.A., Gerchman F., De Felice F.G., McIntyre R.S. (2019). Association between diabetes and mood disorders and the potential use of anti-hyperglycemic agents as antidepressants. Prog. Neuro-Psychopharmacol. Biol. Psychiatry.

[2] Mamakou V., Thanopoulou A., Gonidakis F., Tentolouris N., Kontaxakis V. (2018). Schizophrenia and type 2 diabetes mellitus. Psychiatriki.

[3] Chien I.-C., Chang K.-C., Lin C.-H., Chou Y.-J., Chou P. (2010). Prevalence of diabetes in patients with bipolar disorder in Taiwan: A population-based national health insurance study. Gen. Hosp. Psychiatry.

[4] Chien I.C., Hsu J.H., Lin C.H., Bih S.H., Chou Y.J., Chou P. (2009). Prevalence of diabetes in patients with schizophrenia in Taiwan: A population-based National Health Insurance study. Schizophr. Res..

[5] Abrahamian H., Kautzky-Willer A., Rießland-Seifert A., Fasching P., Ebenbichler C., Kautzky A., Hofmann P., Toplak H. (2019). Mental disorders and diabetes mellitus (Update 2019). Wien. Klin. Wochenschr..

[6] Fleetcroft R., Parekh-Bhurke S., Howe A., Cookson R., Swift L., Steel N. (2010). The UK pay-for-performance programme in primary care: Estimation of population mortality reduction. Br. J. Gen. Pract..

[7] Crawley D., Ng A., Mainous A.G., Majeed A., Millett C. (2009). Impact of pay for performance on quality of chronic disease management by social class group in England. J. R. Soc. Med..

[8] Turner R.C. (1998). The U.K. Prospective Diabetes Study. A review. Diabetes Care.

[9] Wang C.-Y., Yu N.-C., Sheu W.H.-H., Tsai S.-T., Tai T.-Y. (2014). Team care of type 2 diabetes mellitus in Taiwan. Diabetes Res. Clin. Pract..

[10] Hao L.-J., Tien K.-J., Chao H., Hong C.-J., Chou F.-S., Wu T.-J., Chao J.-K., Shi M.-D., Chai K.-L., Ko K.-C. (2011). Metabolic outcome for diabetes shared care program outpatients in a veterans hospital of southern Taiwan. J. Chin. Med. Assoc..

[11] McDaid D., Naylor C., Parsonage M., Knapp M., Fossey M., Galea A. Long-Term Conditions and Mental Health. https://www.kingsfund.org.uk/sites/default/files/field/field_publication_file/long-term-conditions-mental-health-cost-comorbidities-naylor-feb12.pdf.

[12] Columbia Public Health. https://www.publichealth.columbia.edu/research/population-health-methods/repeated-measures-analysis.

[13] Price H.C., Ismail K., Allan B., Castro E., Dashora U., Dhatariya K., Flanagan D., George S., Gregory R., James J. (2018). Royal College of Psychiatrists Liaison Faculty & Joint British Diabetes Societies (JBDS): Guidelines for the management of diabetes in adults and children with psychiatric disorders in inpatient settings. Diabet. Med..

[14] Stenov V., Joensen L.E., Knudsen L., Hansen D.L., Tapager I.W. (2020). Mental Health Professionals Have Never Mentioned My Diabetes, They Don’t Get into That: A Qualitative Study of Support Needs in Adults with Type 1 and Type 2 Diabetes and Severe Mental Illness. Can. J. Diabetes.

[15] Chwastiak A.L., Freudenreich O., Tek C., McKibbin C., Han J., McCarron R., Wisse B. (2015). Clinical management of comorbid diabetes and psychotic disorders. Lancet Psychiatry.

[16] Blixen C.E., Kanuch S., Perzynski A.T., Thomas C., Dawson N.V., Sajatovic M. (2016). Barriers to Self-management of Serious Mental Illness and Diabetes. Am. J. Health Behav..

[17] Chi M.J., Chou K.R., Pei D., Hwang J.S., Quinn L., Chung M.H., Liao Y.M. (2016). Effects and Factors Related to Adherence to A Diabetes Pay-for-Performance Program: Analyses of a National Health Insurance Claims Database. J. Am. Med. Dir. Assoc..

[18] Tan E., Khoo J., Gani L.U., Malakar R.D., Tay T.L., Tirukonda P.S., Kam J.W., Tin A.S., Tang T.Y. (2019). Effect of multidisciplinary intensive targeted care in improving diabetes mellitus outcomes: A randomized controlled pilot study—the Integrated Diabetes Education, Awareness and Lifestyle modification in Singapore (IDEALS) Program. Trials.

[19] Fain J.A., Nettles A., Funnell M.M., Prochownik D.C. (1999). Diabetes Patient Education Research: An Integrative Literature Review. Diabetes Educ..

[20] Hsieh H.-M., Shin S.-J., Tsai S.-L., Chiu H.-C. (2016). Effectiveness of Pay-for-Performance Incentive Designs on Diabetes Care. Med. Care.

[21] Lee T.-T., Cheng S.-H., Chen C.-C., Lai M.-S. (2010). A pay-for-performance program for diabetes care in Taiwan: A preliminary assessment. Am. J. Manag. Care.

[22] Tien K.-J., Hung H.-C., Hsiao J.-Y., Hsu S.-C., Hsin S.-C., Shin S.-J., Hsieh M.-C. (2008). Effectiveness of comprehensive diabetes care program in Taiwanese with type 2 diabetes. Diabetes Res. Clin. Pract..

[23] Hsu C.-C., Tai T.-Y. (2014). Long-term glycemic control by a diabetes case-management program and the challenges of diabetes care in Taiwan. Diabetes Res. Clin. Pract..

[24] Abbate S.L., Brunzell J.D. (1990). Pathophysiology of hyperlipidemia in diabetes mellitus. J. Cardiovasc. Pharmacol..

[25] Guerci B., Ziegler O., Drouin P. (1994). Hyperlipidemia during diabetes mellitus. Recent developments. Presse Médicale.

[26] Riobó Serván P. (2013). Obesity and diabetes. Nutr. Hosp..

[27] Vanderlip E.R., Fiedorowicz J.G., Haynes W.G. (2012). Screening, Diagnosis, and Treatment of Dyslipidemia among Persons with Persistent Mental Illness: A Literature Review. Psychiatr. Serv..

[28] Mazereel V., Detraux J., Vancampfort D., Van Winkel R., De Hert M. (2020). Impact of Psychotropic Medication Effects on Obesity and the Metabolic Syndrome in People with Serious Mental Illness. Front. Endocrinol..

